# Common Errors in Sports Nutrition Meta-Analyses Lead to Distortion of Pooled Effect Estimates. Comment on Viribay et al. Effects of Arginine Supplementation on Athletic Performance Based on Energy Metabolism: A Systematic Review and Meta-Analysis. *Nutrients* 2020, *12,* 1300

**DOI:** 10.3390/nu17142375

**Published:** 2025-07-21

**Authors:** Eric T. Trexler

**Affiliations:** Department of Evolutionary Anthropology, Duke University, Durham, NC 27708, USA; eric.trexler@duke.edu

**Keywords:** arginine, meta-analysis, effect size

## Abstract

Viribay and colleagues published a meta-analysis called “Effects of Arginine Supplementation on Athletic Performance Based on Energy Metabolism: A Systematic Review and Meta-Analysis” in *Nutrients* in May of 2020. This meta-analysis sought to quantify the effects of arginine supplementation on aerobic and anaerobic performance outcomes. In the course of conducting this analysis, the researchers made multiple errors that are common among meta-analyses in the field of sports nutrition. This comment discusses how these issues impact the results and interpretation of the paper, and how individuals who are interpreting or conducting sports nutrition meta-analyses in the future can identify or avoid similar errors.

An article called “Effects of Arginine Supplementation on Athletic Performance Based on Energy Metabolism: A Systematic Review and Meta-Analysis” was published in *Nutrients* in May of 2020 [1]. In this article, Viribay and colleagues sought to summarize the effects of arginine supplementation on both anaerobic and aerobic performance outcomes. The researchers systematically searched the literature and retrieved 18 studies that met the inclusion criteria for their systematic review, with 15 of them warranting inclusion in the quantitative meta-analysis. The results of the meta-analysis indicated that arginine supplementation significantly improved both aerobic (effect size [d] = 0.84; 95% confidence interval: [0.12, 1.56]) and anaerobic performance (d = 0.24 [0.05, 0.43]). As of 8 June 2024, it is the most-cited article from the Special Issue in which it was published. I read this article with great interest and commend the authors for addressing a topic with high potential for practical application. However, upon reviewing the paper, I identified noteworthy observations that warrant correction or clarification.

A recent review paper by Kadlec et al. [2] highlights the most common errors observed in meta-analyses within the field of strength and conditioning. Many of these very same errors are common in sports nutrition meta-analyses, and some of them impact the presently discussed article. One common error involves ignoring outliers. In the presently discussed meta-analysis, the authors include no fewer than three outliers addressing aerobic performance outcomes. These outliers have effect sizes ranging from 3.25 to 5.23, which is far beyond the range typically observed in studies examining even the most effective dietary supplements. Upon closer examination of these outliers, the most likely explanation is that these inflated effect size values are a result of another common error: handling standard errors as standard deviations when calculating standardized mean difference values [2]. The study by Camic et al. [3] reports standard error values instead of standard deviations. Pahlavani et al. [4] report standard deviations of raw values in Table 3 of their article, but appear to mislabel standard error values as standard deviations in Table 4 of their article. These Table 4 values appear to be used for the effect size calculation by Viribay et al. [1], leading to an inflated effect estimate.

When these outliers are maintained within the dataset, the resulting funnel plot suggests troubling statistical characteristics that may preclude the calculation of a pooled effect estimate (Figure 1a). When these outliers are removed, the resulting funnel plot reflects much more acceptable statistical characteristics (Figure 1b) that would enable the calculation of a pooled effect size. Notably, the removal of miscalculated outliers dramatically alters the pooled effect estimate and the overall results of the analysis. Ideally, the corrected analysis would recalculate these three effect estimates using standard deviation values, then assess their individual and collective impact on the pooled effect estimate.

Aside from these three outliers, there are effect sizes included in the analysis that may have been calculated incorrectly. When calculating standardized mean differences, the meta-analyst may utilize the standard deviation values of the baseline and/or post-test group means in the denominator (raw score standardization). Alternatively, they may use the standard deviation values of change scores in the denominator (change score standardization) [5]. While different standardization techniques may be appropriate based on the specific research question being addressed, the resulting effect size metrics have distinct interpretations and should never be combined in the same analysis. It appears that some effect sizes presented in this article are calculated using raw score standardization while others are calculated using change score standardization. If the authors did convert all studies to a uniform effect size metric with a consistent denominator, this procedure is not described within the article.

Furthermore, study-level summary data are not reported with sufficient detail to verify the accuracy of certain effect size calculations. It is unclear how the authors calculated an effect size of 0.00 from the studies by Campbell et al. [6] and Hurst et al. [7], and the time-to-exhaustion effect size reported from the Bailey et al. [8] study appears to compare the arginine group to the citrulline group rather than the placebo group. More transparent and detailed reporting would facilitate more nuanced interpretation of the analysis, with a particular focus on (1) describing any procedures that were used to convert all effect estimates to a uniform effect size metric, and (2) the specific summary data extracted from each study for effect size calculation purposes.

Another common meta-analytic error involves ignoring within-study correlations [2]. When multiple effect sizes from a single study cohort are entered into the same analysis, statistical adjustments should be implemented to account for the dependent nature of these observations and to ensure proper statistical weighting of all studies in the analysis. Defensible remedies include the use of multi-level modeling, the calculation of a single aggregated effect size for each study, or manual adjustment of sample sizes for studies contributing multiple statistically dependent effect estimates. For both the aerobic and anaerobic analyses in the presently discussed paper, as many as four effect sizes from the same study cohort appear to be erroneously treated as independent effect sizes. If any statistical procedure has been used to account for statistical dependency among these values, this procedure is not described within the article.

Meta-analyses play a prominent role in evidence-based practice and are almost universally regarded as the highest tier of the evidence hierarchy. Calculating robust and reliable effect estimates is critical for disseminating accurate information to guide evidence-based practice and to inform the design of adequately powered studies that build on the preceding literature. As such, I am hopeful that the authors of this paper will update their analysis to reflect these corrections, such that researchers and practitioners may utilize a more valid estimate of the typical effect of arginine supplementation when making decisions about research design or practical application. It is also critically important to note that the specific errors observed in the presently discussed paper are neither unique nor rare [2]. As such, this paper serves as an excellent example of common meta-analytic errors that may be instructive for individuals who are conducting or interpreting sports nutrition meta-analyses in the future.

## Figures and Tables

**Figure 1:**
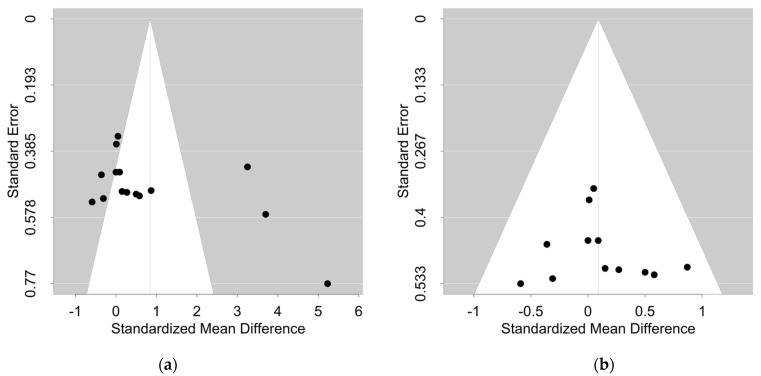
Effects of outlier removal on funnel plot characteristics. (**a**) When outliers are included, the funnel plot characteristics preclude the calculation of a pooled effect estimate. (**b**) When outliers are removed, the funnel plot characteristics are compatible with the calculation of a pooled effect estimate.
